# Large destructive facial hemangioma in PHACE syndrome

**DOI:** 10.4103/0971-9261.57704

**Published:** 2009

**Authors:** N. G. Nagdeve, K. P. Mudkhedkar

**Affiliations:** Pediatric Surgery Unit, Department of Surgery, Government Medical College, Nagpur, India

**Keywords:** Facial hemangioma, infantile hemangioma, PHACE syndrome

## Abstract

We report an infant who presented with large facial hemangioma associated with Dandy-Walker cyst and atrial septal defect. This case is peculiar in that the large facial hemangioma in posterior fossa malformations, hemangiomas, arterial anomalies, coarctation of aorta and other cardiac defects (PHACE) syndrome resulted in massive tissue destruction.

## INTRODUCTION

The acronym PHACE syndrome, first proposed by Frieden,[1] refers to the combination of posterior cranial fossa malformations, hemangiomas, arterial anomalies, coarctation of the aorta or other cardiac defects and eye abnormalities. Large segmental infantile hemangiomas (IH) are most commonly located on the face. The condition is often under recognized, and can easily be confused with Sturge-Weber syndrome. Recent studies estimate that posterior fossa malformations, hemangiomas, arterial anomalies, coarctation of aorta and other cardiac defects (PHACE) syndrome represents about two to three per cent of patients with overall IH and at least 20% of patients with segmental facial IH.[2]

We report an infant with PHACE syndrome which was mistaken as Sturge-Weber syndrome.

## CASE REPORT

**Figure 1:**
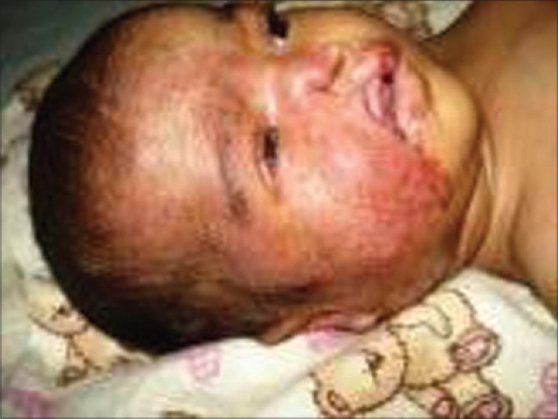
Segmental infantile hemangioma with massive tissue destruction

A two-and-half month old female baby presented with red colored patch over right side of face since birth and progressive ulceration and destruction of right upper lip and right side of nose. On examination, the baby had an erythematous patch mainly on the right side of face extending from forehead down up to angle of mandible and laterally behind the right ear. The lesion crossed the midline and extended on to the left side of nose. On her right side, the upper lip was destroyed resulting in clefting of upper lip. There was a fissuring of angle of mouth on the right. Right alar cartilage and septal cartilage of nose were destroyed. An ophthalmic examination of right side revealed micro-ophthalmos and microcornea with corneal opacity [Figure 1]. Examination of other systems was unremarkable.

**Figure 2:**
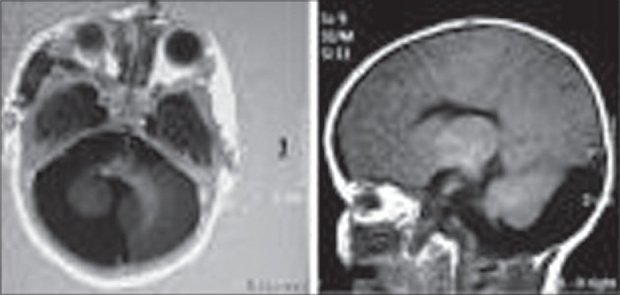
(T1 Weighted Images of MRI Brain): Dandy Walker Cyst Associated with Hypoplastic Vermis and Right Cerebellar Hemisphere

Echocardiography showed osteum secundum type of atrial septal defect (ASD). Doppler studies of carotid and subclavian arteries were normal. Contrast Enhanced computed tomography (CECT) and Magnetic Resonance Imaging (MRI) of brain showed the presence of Dandy Walker cyst associated with hypoplastic vermis and right cerebellar hemisphere [Figure 2]. MR-angiography was normal. The baby was administered oral antibiotics and prednisolone 2mg/kg/day along with oral ranitidine.

Parents were counseled on prognosis and complications of the disease. The baby is under regular follow-up. Soft tissue destruction of face has been halted and the baby is thriving well.

## DISCUSSION

In the PHACE syndrome, apart from facial hemangiomas, structural and vascular anomalies of brain are the most common features (80%), followed by cardiovascular anomalies (33%), ventral developmental defects (25%), and ocular anomalies (20%).[3] Among the CNS abnormalities, Dandy-Walker malformation is most commonly noted while coarctation of aorta is the most common arterial anomaly. The diagnosis of PHACE requires the presence of a typical facial hemangioma in association with only one other congenital anomaly, as affected infants rarely suffer from the complete spectrum.[3] Three systems of our patient were affected i.e. CNS, CVS and right eye.

The PHACE syndrome is uncommon but not rare. It is now recognized that PHACE is probably even more common than Sturge-Weber syndrome; a disorder often confused with PHACE. However, in Sturge-Weber syndrome, the vascular lesion is a slow-flow capillary malformation typically described as port wine stain. It is fully present at birth, shows no signs of proliferation during infancy, and does not regress. Facial capillary malformations are prone to darkening of color and hyperplastic skin changes or gradual hypertrophy of underlying soft tissue and skeleton. Tissue destruction, as observed in our patient, is not a feature of this lesion. But IH, observed in PHACE syndrome, typically shows the growth characteristics of hemangioma. Although majority of the affected infants have an uncomplicated clinical course, a significant subset have function threatening complications, can cause tissue destruction or permanent and disfiguring scars that require medical or surgical interventions.

Typically, hemangiomas seen in PHACE syndrome are of segmental type. These hemangiomas are characterized by a configuration which corresponds with embryological facial prominences.[4] The hemangioma, in the present case, appears to be unique in that though it was mainly present in maxillary segment (segment-2 of Haggstrom *et al.*[5]), it has encroached in all other territories i.e. frontotemporal (segment 1), mandibular (segment 3), and frontonasal (segment 4). It has been suggested that hemangiomas in upper half of the face (segment 1 or segment 4) have a higher risk for associated structural brain, cerebrovascular, and ocular anomalies whereas those involving segment 3 have a greater risk of cardiac defects.[6] This probably explains the association of an extensive IH in present case, with brain, ocular and cardiac anomaly.

Another peculiarity of segmental hemangiomas is their association with complications such as ulceration, birth defects and visceral hemangiomas.[7] IH in this case has resulted in massive tissue destruction halted only by steroid therapy. Thus, segmental IH is more likely to require more intensive and prolonged therapy and have a poorer overall outcome.

To conclude, it is suggested that in all patients with segmental facial IH, PHACE syndrome should be kept in mind and the patient should undergo complete evaluation for all potential anomalies including MRI brain and MR angiography of the head and neck vasculature, echocardiogram and a complete ophthalmologic examination.
